# The Osteogenesis Effect and Underlying Mechanisms of Local Delivery of gAPN in Extraction Sockets of Beagle Dogs

**DOI:** 10.3390/ijms161024946

**Published:** 2015-10-20

**Authors:** Hongcheng Hu, Yinfei Pu, Songhe Lu, Kuo Zhang, Yuan Guo, Hui Lu, Deli Li, Xuefen Li, Zichen Li, Yuwei Wu, Zhihui Tang

**Affiliations:** 1 2nd Dental Center, Peking University School and Hospital of Stomatology, Beijing 100101, China; E-Mails: carefree36@gmail.com (H.H.); puyinfei@yeah.net (Y.P.); tongtong20034@163.com (S.L.); gy2hcp@163.com (Y.G.); cute_luhui@yeah.net (H.L.); leedelee@163.com (D.L.); 2 National Engineering Laboratory for Digital and Material Technology of Stomatology, Peking University School and Hospital of Stomatology, Beijing 100081, China; E-Mail: xflee7@163.com; 3 Department of Laboratory Animal Science, Peking University Health Science Center, Beijing 100191, China; E-Mail: zhangkuo@bjmu.edu.cn; 4 Department of Polymer Science & Engineering College of Chemistry & Molecular Engineering, Peking University, Beijing 100871, China; E-Mail: zcli@pku.edu.cn

**Keywords:** adiponectin, BMP2 (bone morphogenetic protein 2), dental material, mesenchymal stem cells, beagle dog

## Abstract

A plastic and biodegradable bone substitute consists of poly (l-lactic-*co*-glycolic) acid and 30 wt % β-tricalcium phosphate has been previously fabricated, but its osteogenic capability required further improvement. We investigated the use of globular adiponectin (gAPN) as an anabolic agent for tissue-engineered bone using this scaffold. A qualitative analysis of the bone regeneration process was carried out using μCT and histological analysis 12 weeks after implantation. CBCT (Cone Beam Computed Tomography) superimposition was used to characterise the effect of the different treatments on bone formation. In this study, we also explored adiponectin’s (APN) influence on primary cultured human jaw bone marrow mesenchymal stem cells gene expressions involved in the osteogenesis. We found that composite scaffolds loaded with gAPN or bone morphogenetic protein 2 (BMP2) exhibited significantly increased bone formation and mineralisation following 12 weeks in the extraction sockets of beagle dogs, as well as enhanced expression of osteogenic markers. *In vitro* investigation revealed that APN also promoted osteoblast differentiation of primary cultured human jaw bone marrow mesenchymal stem cells (h-JBMMSCs), accompanied by increased activity of alkaline phosphatase, greater mineralisation, and production of the osteoblast-differentiated genes osteocalcin, bone sialoprotein and collagen type I, which was reversed by APPL1 siRNA. Therefore, the composite scaffold loaded with APN exhibited superior activity for guided bone regeneration compared with blank control or Bio-Oss^®^ (a commercially available product). The composite scaffold with APN has significant potential for clinical applications in bone tissue engineering.

## 1. Introduction

We previously established a biodegradable scaffold composed of poly (l-lactic-*co*-glycolic) acid (PLGA), β-tricalcium phosphate (β-TCP) and globular adiponectin (gAPN) [1,2]. This scaffold is particularly attractive for clinical applications due to its excellent biocompatibility and tunable biodegradation rate. The constituents are readily obtainable from synthetic resources in large quantities, and to enhance the functionality of the synthetic materials, peptide or protein ligands such as bone morphogenetic protein 2 (BMP2), which plays a crucial role in osteoinduction [3], can be used for molecular decoration. However, certain issues need to be addressed to facilitate its widespread practical application, including its complicated synthesis, easy degradation, and high cost [3,4,5]. Biological safety questions still need to be coped with, along with issues such as symptomatic ectopic bone formation, bone resorption or remodelling at the recipient region, as well as other possible complications including tumorigenesis and teratogenesis [4,5].

Adiponectin (APN) is the most abundant adipokine in blood plasma; the trimeric form of globular APN (gAPN), results from proteolytic cleavage of full-length APN (fAPN) [6,7]. APN harbors well-characterised insulin-sensitising, anti-inflammatory, and anti-atherosclerotic, as well as anti-diabetic properties [8,9]. More importantly, systemic APN infusion ameliorates diabetic mobilopathy of bone marrow mesenchymal stem cells (BMSCs), lowers glucose concentration, and promotes bone formation in mice with obesity [10]. A wide variety of studies have demonstrated that APN promotes bone formation via several mechanisms, including directly signalling osteoblasts to promote differentiation [11,12], favouring BMSC differentiation toward osteoblastic lineage [13,14,15,16], decreasing sympathetic tone [16,17], and inducing the production of BMP-2 in osteoblasts [18]. However, the role of APN in promoting osteogenic commitment of orofacial bone-derived BMSCs to enhance bone formation has not yet been probed and reported. This constitutes a novel approach for locally promoting bone formation by APN through targeting the osteoblasts and osteoclasts simultaneously.

We conducted a comparative study of osteogenesis in scaffolds loaded with BMP2, gAPN, or Bio-Oss^®^ (a commercially available product that is routinely and successfully used in surgical dentistry and orthopaedic surgery), through the placement of biomaterials in the fresh extraction sockets of beagle dogs. We also experimentally investigated how gAPN affects osteoblast differentiation of human jaw bone marrow mesenchymal stem cells (h-JBMMSCs) *in vitro*. We hypothesised that scaffolds loaded with gAPN would enhance bone formation through promoting osteogenic commitment of h-JBMMSCs.

## 2. Results

### 2.1. Characterization of Bone Substitutes Loaded with gAPN or BMP2

**Figure 1:**
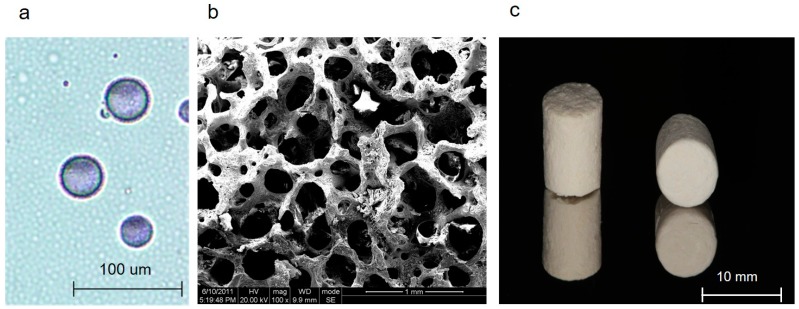
Optical microscopic (Nikon, Berlin, Germany) image ((**a**) scale bars = 100 µm) of chitosan microspheres with gAPN (blue purple); SEM image of the β-TCP scaffold ((**b**) scale bars = 1 mm, particle size 0.25–1 mm, pore size 100–600 μm) prepared in the PLGA (poly l-lactic-*co*-glycolic acid) scaffold. The appearance of PLGA scaffold ((**c**) scale bars = 10 mm).

Figure 1a demonstrates the morphology of chitosan microspheres with gAPN. The average diameter was 18.59 ± 15.20 µm (Figure 1a). The microstructure of the porous β-TCP is shown in Figure 1b (pore size 100–600 μm). The appearance of PLGA scaffold was a block mass (Figure 1c).

### 2.2. Clinical Evaluation

**Figure 2:**
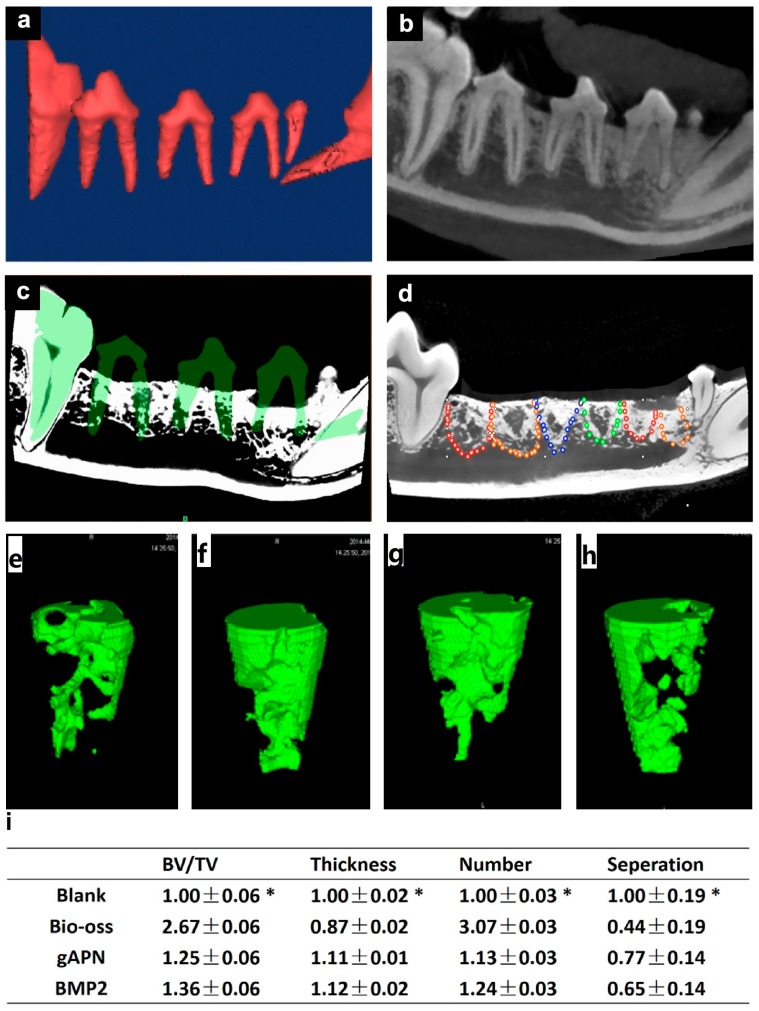
Extraction socket location and μCT analysis: (**a**) 3D CBCT (Cone Beam Computed Tomography) reconstructions and (**b**) the teeth used for reference; (**c**) Superimposition 3D CBCT and μCT models; (**d**) Highlighted borderline of the extraction socket in the μCT models (red: gAPN-treated group, orange: BMP2-treated group, blue: Bio-Oss group, green: blank group). Representative 3D reconstruction of the trabecular structure in the extraction socket: (**e**) Blank; (**f**) Bio-oss; (**g**) gAPN and (**h**) BMP2; and (**i**) Changes in trabecular bone parameters, including trabecular bone volume normalised by tissue volume (BV/TV), trabecular thickness (Tb.Th), trabecular number (Tb.N), and trabecular separation (Tb.Sp) are shown as means ± SD, with *n* = 4. * *p* < 0.05, as determined by one-way ANOVA.

All sites healed without complications, and were observed daily during routine post-surgery checks. We observed full coverage of each site with a slightly inflamed mucosa during removal of the sutures after two weeks. At the time of sacrifice (12 weeks), all extraction sites were covered by an apparently non-inflamed keratinised ridge mucosa, and the outline of the extraction socket were difficult to identify. The socket distribution and bone fill were observed radiographically using the superimposed models, as shown in Figure 2.

### 2.3. Bone Substitutes Loaded with gAPN or BMP2 Promoted Bone Formation

**Figure 3:**
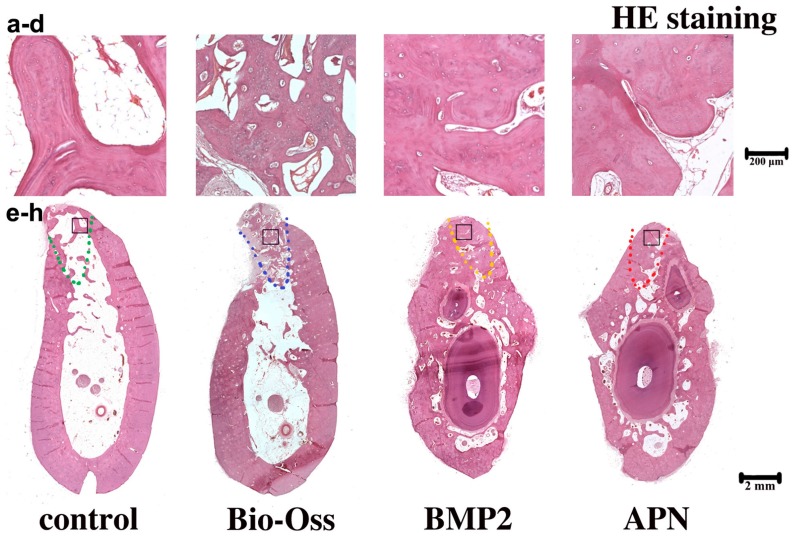
Decalcified H&E staining of the extraction sockets following the different 12-week treatments: (**a**)The magnifying control group; (**b**) Bio-Oss group; (**c**) BMP2 group; (**d**) APN group; (**e**–**h**) represent the integral extraction socket of control group (**e**); Bio-Oss group (**f**); BMP2 group (**g**); APN group (**h**). The black square represents the magnifying area. The dashed line represents the extraction sockets. Magnification: **upper panel** 40×; **lower panel** 4×. *n* = 4 for each group.

To investigate the composites loaded with gAPN or BMP2 in bone metabolism, we delivered gAPN or BMP2 via the PLGA scaffold into the dogs’ extraction sockets. We then used μCT (see Figure 2) and hematoxylin and eosin (H&E) staining (see Figure 3) to analyse changes in the trabecular bone (Tb) structure and mineral density following 12 weeks of scaffold placement. Compared with blank controls, after 12 weeks in extraction sockets, the gAPN and BMP2 scaffolds led to a significant improvement in the trabecular bone volume (BV), trabecular thickness (Tb.Th), trabecular number (Tb.N), and to a reduction in the trabecular separation (Tb.Sp) distance (see Figure 2i). However, the Bio-Oss group exhibited the largest ratio of trabecular BV normalised by tissue volume (BV/TV) and Tb.N, as well as the lowest Tb.Sp (see Figure 2i). H&E staining further demonstrated the ability of gAPN and BMP2 (see Figure 3) scaffolds to increase the trabecular bone mass and trabecular separation of the extraction sockets. No major bone formations were evident in the central region of the extraction sockets in the control group (see Figure 3).

As shown in Figure 3, all extraction sockets demonstrated trabecular bone fill with a more or less developed bone marrow after eight weeks healing. The anorganic bovine bone was with surrounded by lamellar bone and granulation tissue in the Bio-Oss group. In contrast, the extraction sockets treated with scaffolds loaded with gAPN or BMP2 exhibited pronounced resorption over the eight weeks and the newly formed trabecular bone apically and laterally extended from the socket walls, occupying almost the entire socket.

**Figure 4:**
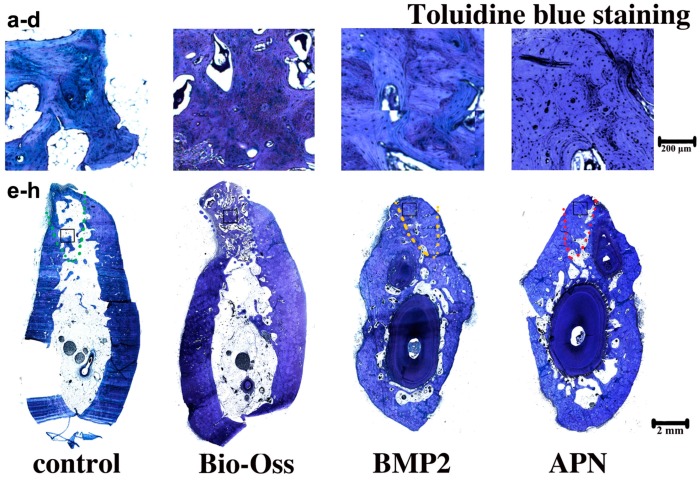
Decalcified toluidine blue staining of extraction sockets following the different 12-week treatments: (**a**)The magnifying control group; (**b**) Bio-Oss group; (**c**) BMP2 group; (**d**) APN group; (**e**–**h**) represent the integral extraction socket of control group (**e**); Bio-Oss group (**f**); BMP2 group (**g**); APN group (**h**). The black square represents the magnifying area. The dashed line represents the extraction sockets. Magnification: **upper panel** 40×; **lower panel** 4×. *n* = 4 for each group.

Compared with the control and Bio-Oss groups, the extraction sockets treated with scaffolds loaded with gAPN or BMP2 exhibited increase in the new bone formation, as shown by the increased toluidine blue staining images in Figure 4. We observed abundant newly formed osteoid in the supposed root area; however, in the Bio-Oss group the osteoid exhibited closer proximity and contact with the demineralised bovine granule, as shown by the slightly lighter staining than the osteoid. Thus, the new bone formation in the gAPN or BMP2 loaded group was superior to that of the control and Bio-Oss group (Figure 4).

**Figure 5:**
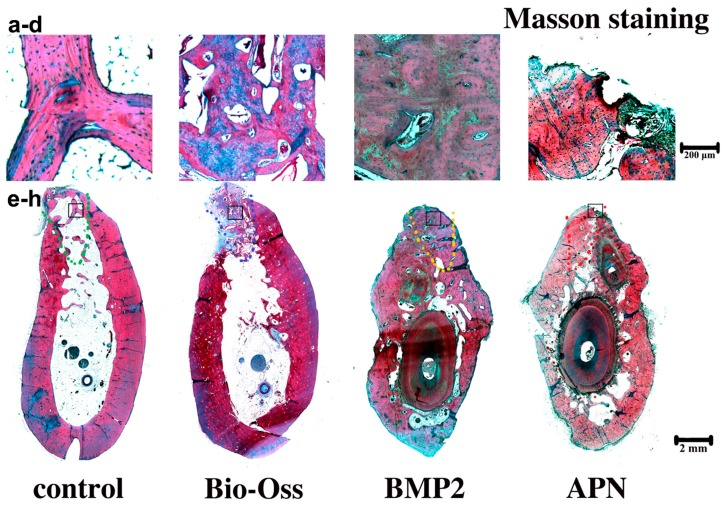
Decalcified toluidine Masson staining of extraction sockets following the different 12-week treatments: (**a**) The magnifying control group; (**b**) Bio-Oss group; (**c**) BMP2 group; (**d**) APN group; (**e**–**h**) represent the integral extraction socket of control group (**e**); Bio-Oss group (**f**); BMP2 group (**g**); APN group (**h**). The black square represents the magnifying area. The dashed line represents the extraction sockets. New new bone is shown in red. Magnification: **upper panel** 40×; **lower panel** 4×. *n* = 4 for each group.

Masson staining was also used to observe bone formation. In the blank control, only a thin layer of lamellar trabecular was observed, and the largest area was bone marrow, as shown in Figure 5a. Newly formed trabecular was observed hailed from the edges of the extraction socket toward the central area in the control group. In this histological sections, only a small volume of blue ossein was distributed on the new bone surface, and less was observed than that in host bone. In the Bio-Oss group, the central regions were mainly occupied by mineral portions of bovine bone. The implanted bovine bone had not been replaced, and the scaffold surface was surrounded by little osteoid, as shown in Figure 5b. In the gAPN and BMP2 treatment groups, the implanted scaffold had been replaced, and the treated defect was almost covered by mature lamellar bone, as shown by the red staining, and the blue ossein was spreaded consistently in the new bone matrix (see Figure 5c,d). These results further demonstrate that the gAPN and BMP2 effectively enhanced the trabecular bone density and mineralisation after 12 weeks of socket placement.

### 2.4. gAPN or BMP2 Treatment Increased Osteoblast Differentiation

**Figure 6:**
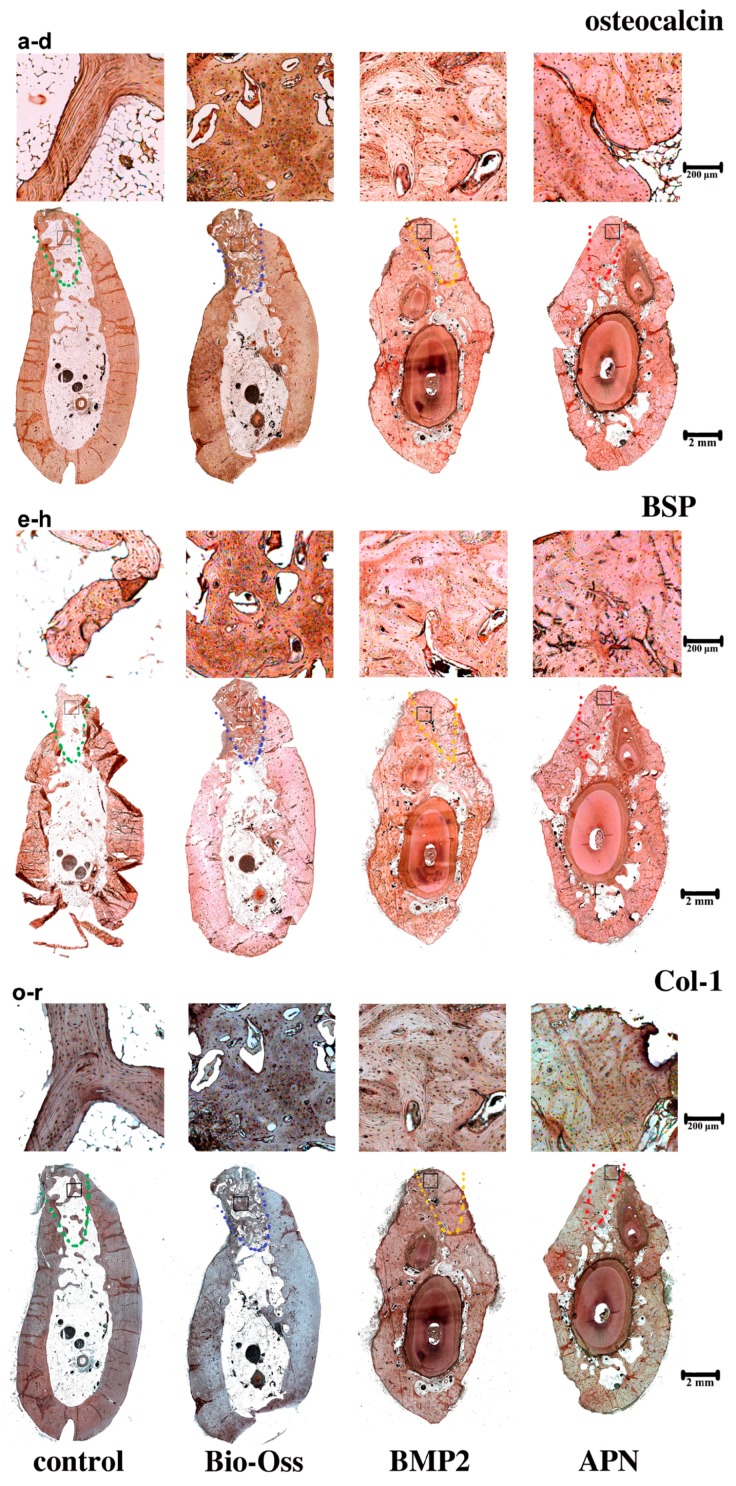
Expression of osteogenic related protein following the different 12-week treatments in the extraction sockets. Imunohistochemical analysis of extraction socket trabecular bone sections for (**a**–**d**) OCN (Osteocalcin); (**e**–**h**) BSP (Bone sialoprotein) and (**o**–**r**) Col-1 (Collagen 1). (**a**,**e**,**o**) the control group; (**b**,**f**,**p**) Bio-Oss group; (**c**,**g**,**q**) BMP2 group; (**d**,**h**,**r**) APN group. The black square represents the magnifying area. The dashed line represents the extraction sockets. Magnification: **upper panel** 40×; **lower panel** 4×. *n* = 4 for each group.

We characterised the expression in osteoblast-related genes by immunostaining. The extraction sockets treated with gAPN and BMP2 exhibited significantly increased osteocalcin and BSP expression compared with the control group, as shown in Figure 6a,b, Table S1. The expression of collagen type-I, which is a key scaffold and template for mineral formation [19], was significantly increased in the gAPN or BMP2 groups compared with the control, as shown in Figure 6c,d, Table S1. The expression of collagen in Bio-Oss group, however, was lower than in the other three groups.

### 2.5. gAPN Promoted Osteogenic Differentiation of h-JBMMSCs (Human Jaw Bone Marrow Mesenchymal Stem Cells)

**Figure 7:**
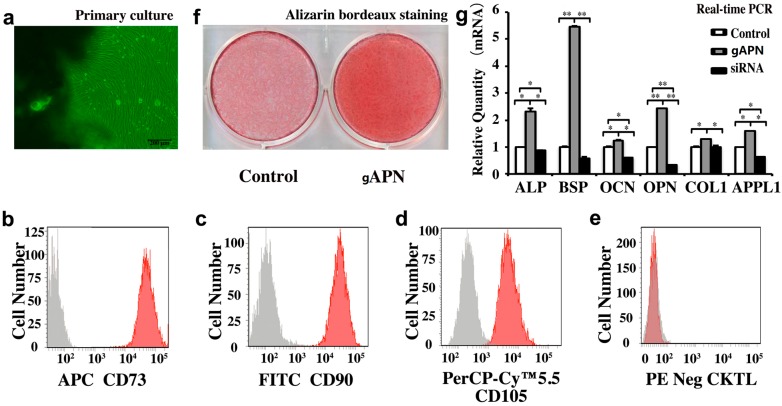
gAPN promoted the osteogenic commitment of h-JBMMSCs to favour osteoblastic differentiation. (**a**) The primary cultures were explanted from human granulation tissue in the 3rd molar extraction socket; (**b**–**e**) The primary cultured h-JBMMSCs were identified by flow cytometry. The h-JBMMSCs were positive for CD73, CD90, and CD105, but were negative for CD34, CD11b, CD19, CD45, and HLA-DR (red). All the results were with h-JBMMSC (red) and isotype control (light gray); (**f**) h-JBMMSCs were treated with vehicle and gAPN in osteogenesis induction medium for 21 days, followed by ascorbic acid staining; (**g**) Changes in gene expression of h-JBMMSCs following 24 h of gAPN treatment analysed using quantitative RT-PCR. Data are shown as the mean ± SD, with *n* = 4. * *p* < 0.05, ** *p* < 0.01 as determined by one-way ANOVA.

h-JBMMSCs have the potential to differentiate into osteoblasts, chondrocytes, and adipocytes [20]. We found that the administration of gAPN promoted bone formation in extraction defects more actively than in the control and Bio-Oss groups, so we investigated the role of gAPN in regulating osteogenic commitment of h-JBMMSCs. The identified h-JBMMSCs were treated with 1 µg/mL of gAPN, and osteogenesis was evaluated using alizarin bordeaux staining, as shown in Figure 7a,b. We observed more positive alizarin bordeaux staining compared with control cells in the gAPN-treated cells, as shown in Figure 7c. Additionally, mRNA levels of ALP, BSP, Col-1, OCN, and OPN were significantly enhanced in the gAPN-treated cells compared with the control, as shown in Figure 7d.

To further explore the mechanisms underlying the gAPN-induced osteogenic differentiation of the h-JBMMSCs, we evaluated the levels of APPL1 expression. We found that APPL1 expression increased significantly following gAPN treatment compared with the untreated control, as shown in Figure 7d. Additionally, we pretreated the h-JBMMSCs with the specific APPL1 siRNA (see Figure 7d), and found that downregulation of APPL1 resulted in downregulation of ALP, BSP, Col-1, OCN, and OPN.

## 3. Discussion

### 3.1. Superimposition of CBCT (Cone Beam Computed Tomography) Models for the Location of Extraction Sockets

Conventional X-ray [21], gravimetry [22], extraoral (casts) or intra-oral exams [23,24], optical projection [25], optical scanning [26], measurements on photographs [27], and bone mapping via sounding [24] cannot accurately pinpoint the 3D location of extraction sockets, because of artefacts including dimensional changes in impression and cast materials, access limitation, and saliva influence.

This is the first study to use CBCT superimposition to locate extraction sockets (see Figure 2a,b). CBCT scans are a mature 3D virtual tool for diagnosis, treatment planning, and pre-clinical simulations. One single scan by CBCT can provide not only detailed 2D images of the root socket structures in axial, coronal, and sagittal slices, but also a 3D reconstruction of the jaw. The 3D models of the extraction sockets were visible clearly and segmented easily from the CBCT scans. 3D models of the same extraction sockets in different time points could be superimposed using common stable landmarks as reference to evaluate the changes of extraction sockets [28,29]. We chose the cuspid of the canine and the first molar as the superimposition references, because the locations of these teeth are stable in mature beagles, and were not expected to change following the operation.

### 3.2. Local Administration of gAPN Promoted Trabecular Bone Formation in Extraction Sockets

After precisely locating the extraction sockets, we used μCT (see Figure 2) and histological analysis (see Figure 3, Figure 4 and Figure 5) to analyse changes in the trabecular bone structure and mineral density following three months of scaffold placement loaded with gAPN or BMP2. We found that the gAPN and BMP2 scaffolds significantly increased TV/BV, Tb.Th, and Tb.N, and resulted in a decrease in the Tb.Sp separation (see Figure 2i). The control group exhibited decreased bone mineralisation and density, and enlarged bone marrow area, compared with the gAPN and BMP2 groups (see Figure 2i). Histological analyses of trabecular bone demonstrated the function of the gAPN and BMP2 scaffolds to increase the trabecular bone and reduce the marrow area in extraction sockets after 12 weeks compared with the control group (see Figure 3, Figure 4 and Figure 5).

The Bio-Oss group exhibited the largest values of BV/TV, Tb, and Tb.N, as well as the smallest Tb.Sp (see Figure 2i); however, toluimide blue staining revealed the lowest formation of new bone in the BIO-Oss group (see Figure 4b). This was consistent with the results of Masson staining (see Figure 5b). In micro-CT experiments, all the mineralized bone could be detected, included anorganic bovine bone (Bio-Oss). This was the limitation of the micro-CT methodology. In the Bio-Oss group, the central regions were mainly occupied by mineral portions of bovine bone (Figure 3b,f) and less new bone formation (Figure 4b,f and Figure 5b,f). In the gAPN and BMP2 treatment groups, the implanted scaffold had been replaced by new bone (Figure 3g,h). So, the Bio-Oss group had more dense bone in micro-CT measurement. However, histology analysis experiments could distinguish the bone substitute and new bone formation. Compared with the Bio-Oss groups, the extraction sockets treated with scaffolds loaded with gAPN or BMP2 exhibited more toluidine blue (Figure 4) and Masson staining (Figure 5), clearly indicating the new bone formation in gAPN or BMP2 group. This is the reason that the trabecular separation is low (more dense) by CT evaluation while new bone formation is low by histology evaluation in Bio-Oss group. Our results are in line with previous studies that BMPs are potent osteoinductive factors [30], and can regulate the function and differentiation of cells involved in bone formation and healing [31,32]. This article is the first to report that scaffolds loaded with gAPN exhibit osteoinduction, which is consistent with the retarded growth of bone explants in APN-KO mice [33], and the observation that AdipoR1 mice exhibit increased bone volume and trabecular numbers than wild-type mice [11].

### 3.3. Local Placement of Scaffold Loaded with gAPN Increased Osteoblast Differentiation and Collagen Type I Expression

We explored changes in osteoblast-related protein expression by immunostaining socket slides of the different groups 12 weeks after scaffolds implantation. The gAPN and BMP2 groups exhibited significantly increased numbers of osteoblasts expressing OCN and BSP compared with the control (see Figure 6a,b, Table S1). However, the expression of collagen type I was lowest in the Bio-Oss group (see Figure 6c, Table S1), which is consistent with the lower intensity of toluidine blue and Masson staining in the Bio-Oss group (see Figure 4 and Figure 5). These findings indicate that the scaffolds loaded with gAPN or BMP2 exhibited superior osteoinduction compared with Bio-Oss.

### 3.4. Osteogenic Differentiation of h-JBMMSCs Induced with gAPN in Vitro

Bone remodelling is a delicate balance between bone resorption by osteoclasts and bone deposition by osteoblasts [34]. This equilibrium is influenced by differentiation and activity of osteoclasts and osteoblasts and [35]. To gain insight into the mechanisms underlying gAPN-induced oestrogenic differentiation of h-JBMMSCs, we investigated the expression of osteogenesis-related genes after 24 h of exposure to gAPN. We found that gAPN promoted osteogenic differentiation of the primary cultured h-JBMMSCs *in vitro*, which was accompanied by greater mineralisation deposition activity (see Figure 7c), and greater expression of osteoblast-related genes such as collagen type I, osteopontin (OPN), bone sialoprotein (BSP), and osteocalcin (OCN), and which was reversed by APPL1 siRNA (see Figure 7d). Many of the effects of APN in various cell types are known to be mediated by APPL1 [36,37], but this is the first demonstration of such a mechanism in h-JBMMSCs. APPL1 is involved in cell signalling by activating Akt, PI3-kinase (PI3K), the adiponectin receptor, and TrkA [38]. Previous studies have shown that PI3K [36], Akt [37], and TrkA [39] pathways affect osteoblast function via the proliferation and differentiation of BMSCs. More work is needed to investigate the involvement of APN and its signalling pathways in regulating h-JBMMSCs *in vivo*.

## 4. Experimental Section

### 4.1. Recombinant gAPN

The His-tagged C-terminal part of human APN (*i.e.*, amino acids 106–244) was expressed in BL21 (DE3) bacterial cells. His-tagged gAPN was purified using GE Pharmacia AKTA Purifier 10 (Ramsey, MN, USA) followed by endotoxin removal (L00338, GenScript, Piscataway, NJ, USA) and Zeba Spin Desalting Columns (89893, Pierce, Rockford, IL, USA). The gAPN solution was filtrated to remove bacteria using a 0.22-µm separating film.

### 4.2. Materials

Chitosan (with a molecular weight of 500 kDa and a deacetylation grade of 90%), span-80 and tripolyphosphate (TPP) were purchased from Beijing Chemical Reagents Company (Beijing, China). PLGA (nLA/nGA = 50/50) was obtained from the Jinan Daigang Bioengineering Co., Ltd. (Jinan, Shandong, China), and β-TCP was prepared in our laboratory (particle size 0.25–1 mm). The other reagents were of analytical grade, and applied in accordance with the manufacturer’s instructions. All chemicals were sterilised using cobalt-60 γ radiation before use.

### 4.3. Preparation of Scaffolds

Chitosan microspheres were formulated using an emulsion-ionic modus [40]. In brief, 1 mL of 2% (*v*/*v*) aqueous acetic acid with 1 mg gAPN or 0.2 mg BMP2 (Sigma-Aldrich, St. Louis, MO, USA) was added to a mixture, which was consist of 900 mg of chitosan dissolved in 29 mL of 2% (*v*/*v*) aqueous acetic acid. Then, the hybrid was put into 300 mL of liquid paraffin include 2% (*w*/*v*) of span-80 and mechanically mixed for 2 h (C-MAG HS 10, IKA^®^ Works Inc., Wilmington, NC, USA). Next, 70 mL of 5% (*w*/*v*) TPP was put into the emulsion and mixed for 4 h at room temperature. Excess petroleum ether, isopropyl alcohol, and distilled water were used to wash the mixture repeatedly, then the microspheres were acquired with lyophilisation. Microsphere-scaffold composites were fabricated by thermal phase separation. Then, 12 mL of 1,4-dioxane was used to dissolve 720 mg of PLGA, and 360 mg of β-TCP was put into the mixture after mixing for 30 min.

The β-TCP were dispersed by ultrasonication (Huanan Ultrasonic Equipment Co., Ltd., Guangzhou, China) for 10 min. Next, 240 mg of the chitosan-gAPN or chitosan-BMP2 microspheres was added to the solution, which was then stirred to completely disperse the microspheres and finally frozen using liquid nitrogen. The PLGA/β-TCP/CMs scaffolds with and without APN or BMP2 were acquired after having been lyophilised according to the manufacturer’s instruction (Labconco, Kansas City, MO, USA). The above procedures were conducted under aseptic conditions.

### 4.4. Surgical Procedure

**Figure 8:**
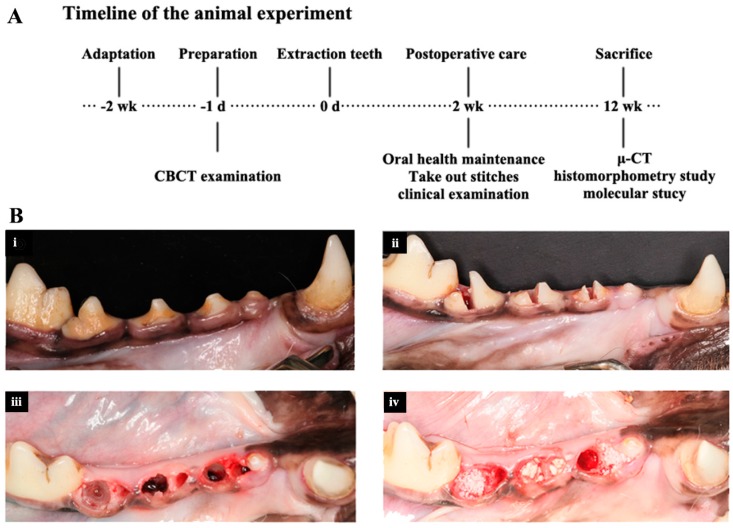
(**A**) Timeline of the treatment; (**B**) Treatment groups. (**i**) The 2nd, 3rd, and 4th premolar teeth before extraction; (**ii**) Teeth were segmented before extraction; (**iii**) Extraction sockets immediately following removal of the roots; extraction was accomplished with as little trauma to the alveolus as possible; (**iv**) The control, Bio-Oss, and scaffolds loaded with BMP2 or APN were randomly applied to the extraction sockets (lateral view of the left jaw).

We used male beagle dogs aged 14–18 months with body weights of 12–15 kg. All animals had intact permanent dentition and healthy periodontium. All the operations were done after a 2-week adaptation, as shown in Figure 8. Throughout the experiments, the dogs were fed once per day with soft food to prevent mechanical trauma during healing. All protocols involving the dogs met the guidelines described in the Association for Assessment and Accreditation of Laboratory Animal Care and were approved by the Peking University Health Centre Institutional Animal Care and Use Committee and Peking University Health Centre Ethnics Committee (LA2013-3, January 2013). One professional surgeon performed all operations.

The 2nd, 3rd, and 4th premolar teeth in the unilateral mandible were extracted according to the protocol described in Reference [41]. During surgery, the animals were anaesthetised with intramuscularly administered atropine (0.04 mg/kg) and ketamine (2.5 mg/kg), as well as a 4% solution of intravenously administered pentobarbital sodium (25–30 mg/kg). Before extraction of the teeth, local anaesthesia with 2% xylocaine (1/80,000 epinephrine) was administrated. The same medications were used to obtain the pre-surgery CBCT.

### 4.5. Ridge Preservation

The 12 fresh extraction sockets were randomly allocated to blank control (I), Bio-Oss (0.25–1 mm particle size, Geistlich, Wolhusen, Switzerland) (II), scaffolds loaded with BMP2 (III) or APN (IV). The choice of the different treatments was determined using casting lots. Mucoperiosteal flaps were raised with periosteal-releasing incisions to make sure the wound could be closed without any tension, and then the wounds were closed with interrupted and mattress sutures, as shown in Figure 8. Antibiotic therapy (ampicillin sodium; 25 mg/kg intramuscularly daily) and analgesics (naproxen; 1.1 mg/kg taken orally) were administered for 5 days, and the sutures were removed 15 days after the surgery. The euthanasia was performed by anesthesia overdose 12 weeks after scaffolds implantation.

### 4.6. CBCT Scans

CBCT scans were taken 1 day prior to extraction of the teeth using a NewTom VG 3D Imaging System (NewTom Dental Inc., Verona, Italy) with a window of scan of 12 cm × 8 cm and a voxel size of 0.15 mm and exported data using the Digital Imaging and Communications in Medicine (DICOM) format.

### 4.7. μCT Scans

Radiographs of the trabecular bone architecture of dog mandibles were obtained using a high-resolution µCT system (Inveon, Siemens, Germany) following sacrifice after 12 weeks. The specimens were scanned at an operating voltage of 60 kV and a current of 300 μA, with an effective pixel size of 8.5 μm. Data from the µCT scans were exported using DICOM format.

### 4.8. Socket Location and μCT Analysis

Three-dimensional (3D) STL models of the premolars and 1st molars were segmented from the CBCT DICOM data prior to extraction of the teeth using Materialise Mimics 14.1 (an interactive medical image control system, Materialise Inc., Leuven, Belgium). The canines and 1st molars were segmented using the same software from the corresponding μCT DICOM files. The first molar and the canine were used as reference objects. The 3D STL models before extraction were superimposed and repositioned onto the 3D STL models from the μCT data using digital image process software (Geomagic Studio 2012, Raindrop Geomagic, Research Triangle Park, Durham, NC, USA) The repositioned 3D digital model prior to extraction, which contained information on the position of the extracted teeth, was used as a mask to help locate the extraction sockets, as shown in Figure 2c, and to locate the region of interest (ROI). A root-shaped region was dissected. The threshold of trabecular bone was the same for each specimen (as shown in Figure S1). Additionally, BV/TV, trabecular number (Tb.N), trabecular thickness, and Tb.Sp were analysed using software provided by Materialise, as shown in Figure 2i.

### 4.9. Histomorphometry Analysis

15% EDTA was used to demineralise sample. Then, the samples were processed by graded alcohols, xylene and paraffin. The thickness of slices was 4-μm. To quantify the new bone formation, three pieces of histological slices were stained with H&E, toluidine blue, and Masson, and randomly selected and observed using an optical microscope at magnifications of 20 and 200.

### 4.10. Immunohistochemical Labelling

A molecular analysis of the bone regeneration process was carried out using immunohistochemistry 12 weeks after scaffolds implantation in the extraction sockets of Beagle dogs. For immunohistochemistry, all tissue slices were deplasted and treated with antibodies to determine the expression of collagen type I (Col-1) (1:100, Santa Cruz, CA, USA), osteocalcin (OCN) (1:100, Santa Cruz, CA, USA), and bone sialoprotein (BSP) (1:100, Santa Cruz, CA, USA).

### 4.11. Numerical Evaluation of the Histology

The results of immunohistochemistry staining were semiquantitatively valued by two researchers. The researchers did not know the details of specimens. The intensity of staining (strong, moderate, weak, and negative) was graded as 4, 3, 2 and 1, respectively. The final labeling index obtained by the previous intensity results multiplied the percentage of positive cells (0, 25%, 50% or 75%).

### 4.12. Donor Socket Granulation Tissue and Primary h-JBMMSCs Cultures

Human mandible third molars were extracted and the granulation bone marrow was gained from the socket. Primary h-JBMMSCs were established from the granulation tissue explants of donors using a method described previously [20]. In brief, the granulation tissue was minced and placed separately into the 12-well plates. The cell culture medium was changed per 2–3 days following the emergence of outgrowth. The cells were cultured in medium consisting of α-MEM (Life Technologies, Rockville, MD, USA), 15% foetal bovine serum (FBS, Life Technologies, Rockville, MD, USA), 100 µM of l-ascorbic acid 2-phosphate (WAKO, Tokyo, Japan), 2 mM of l-glutamine (Life Technologies), 100 U/mL of penicillin, and 100 µg/mL of streptomycin (Sigma-Aldrich, St. Louis, MO, USA) at 37 °C in 5% CO_2_. The study protocol was documented by the Medical Ethical Commission of the Peking University School and Hospital of Stomatology, Beijing, People’s Republic of China (PKUSSIRB-201520026). All patients provided informed consent. Human tissue samples were handled according to the tenets of the Declaration of Helsinki.

### 4.13. Flow Cytometry

The cells were first dissociated and followed by process with the antibodies: FITC Mouse Anti-Human CD90 (BD); APC Mouse Anti-Human CD73 (BD); PerCP-Cy™ 5.5 Mouse Anti-Human CD105 (BD); PE hMSC Negative Cocktail including CD34 PE, CD11b PE, CD19 PE, CD45 PE, HLA-DR PE (BD); and the respective isotype-matched negative control antibodies on ice for 30 min. The cells were analysed using a MACSQuant Analyzer and the FlowJo software package (Tree Star, Ashland, OR, USA) were used to assess the data.

### 4.14. h-JBMMSCs Osteogenic Induction

Cells from the fourth passage were choosed to conduct the experiments. To investigate cell osteogenic differentiation, cells were seeded at 2 × 10^4^ per well in 12-well plates. After the cultures reached confluence, the medium was changed to DMEM with 2% (*v*/*v*) KnockOut™ Serum Replacement, 100 U/mL penicillin, and 100 mg/mL streptomycin containing 10-nM dexamethasone, 10-mM β-glycerophosphate, and 50-μg/mL l-ascorbic acid (Sigma-Aldrich, St. Louis, MO, USA). The cells were then further cultured for 21 days with or without 1-μg/mL APN.

### 4.15. Knockdown of APPL1 in h-JBMMSCs

siRNA sequences (Santa Cruz, CA, USA) designed for the knockdown of human APPL1 were tested with h-JBMMSCs. Because it is difficult to transfect stem cells, Lipofectamine^®^ 2000 Transfection Reagent was used. The h-JBMMSCs reached 30%–50% confluence in 6-well plates, and transfected with 100-nM scrambled or APPL1 siRNA for 6 h using Lipofectamine^®^ 2000 Transfection Reagent. The siRNA sequence was GCUUAGUUCUUGUCAUGCAtt. APN treatment commenced 48 h post-transfection, and the APPL1 knockdown efficiency was assessed using the real-time PCR described below.

### 4.16. Quantitative Real-Time PCR

TRIzol (Invitrogen, Carlsbad, CA, USA) was used to extract sample total RNA. SuperScript III reverse transcriptase (Life Technologies) and oligo(dT)20 primer (Life Technologies) were used to generate the first strand of cDNA. Power SYBR Green PCR Master Mix (Applied Biosystems) and a 7500 Real-Time PCR System (Applied Biosystems) were used to perform Real-time PCR. β-actin served as a control. The sequence of primers was as follows: human β-actin sense 5′-CAAGGCCAACCGCGAGAAGATGAC-3′, antisense 5′-GCCAGAGGCGTACAGGGATAGCACA-3′; ALP sense 5′-CCCGCTTTAACCAGTGCAAC-3′, antisense 5′-GAGCTGCGTAGCGATGTCC-3′; BSP sense 5′-GGCCACGATATTATCTTTACAAGC-3′, antisense 5′-CCTCTTCTGAACTGTCATCTCCA-3′; Col-1 sense 5′-GAGGGCCAAGACGAAGACATC-3′, antisense 5′-CAGATCACGTCATCGCACAAC-3′; OCN sense 5′-GGCGCTACCTGTATCAATGG-3′, antisense 5′-GTGGTCAGCCAACTCGTCA-3′; OPN sense 5′-GAAGTTTCGCAGACCTGACAT-3′, and antisense 5′-GTATGCACCATTCAACTCCTCG-3′.

### 4.17. Statistical Analysis

Data were shown as the mean ± standard deviation (SD). One-way analysis of variance (ANOVA) was used to evaluated the statistically significant differences (*p* < 0.05) among the various groups. SPSS 19.0 software package (SPSS Inc., Chicago, IL, USA) was used for all statistical analysis.

## 5. Conclusions

We conducted an *in vivo* investigation of the extraction sockets of beagles, and found that composite scaffold loaded with APN exhibited superior activity for guided bone regeneration compared with blank control. *In vitro* experiments revealed that 1 μg/mL of gAPN can induce osteoblastic differentiation in human h-JBMMSCs. The composite scaffold with APN has significant potential for clinical applications in bone tissue engineering.

## Supplementary Materials

Supplementary materials can be found at http://www.mdpi.com/1422-0067/16/10/24946/s1.

## Abbreviations

gAPN: globular adiponectin; PLGA: poly (l-lactic-*co*-glycolic) acid; β-TCP: β-tricalcium phosphate; CBCT: cone beam computed tomography; BMP-2: bone morphogenetic protein 2; h-JBMMSCs: human jaw bone marrow mesenchymal stem cells; Tb: trabecular bone; BV: bone volume; TV: tissue volume; Tb.Th: trabecular thickness; Tb.N: trabecular number; Tb.Sp: trabecular separation; OCN: osteocalcin; OPN: osteopontin; BSP: bone sialoprotein; Col-1: Collagen Type 1; BMSCs: bone marrow mesenchymal stem cells; TPP: tripolyphosphate; ROI: region of interest; FBS: foetal bovine serum; SiRNA: small interfering RNA.
