# Supplementary material for: The Osteogenesis Effect and Underlying Mechanisms of Local Delivery of gAPN in Extraction Sockets of Beagle Dogs
Source: Int J Mol Sci. 2015 Oct 20;16(10):24946–64. doi: 10.3390/ijms161024946 (PMC4632783; doi:10.3390/ijms161024946)
Supplement: Supplementary file 1 [file ijms-16-24946-s001.pdf]

Supplementary Information

Table S1. Expression level of Osteocalcin, BSP and Col-1 in the socket sections of the different groups

Target Protein	Control	Bio-Oss	BMP2	APN
Osteocalcin	1 ± 0.25	2.1 ± 0.69	5.8 ± 0.30	5.9 ± 0.27
BSP	1 ± 0.27	2.6 ± 0.32	4.0 ± 0.47	4.1 ± 0.11
Col-1	1 ± 0.27	1.9 ± 0.35	5.8 ± 0.98	5.6 ± 0.92

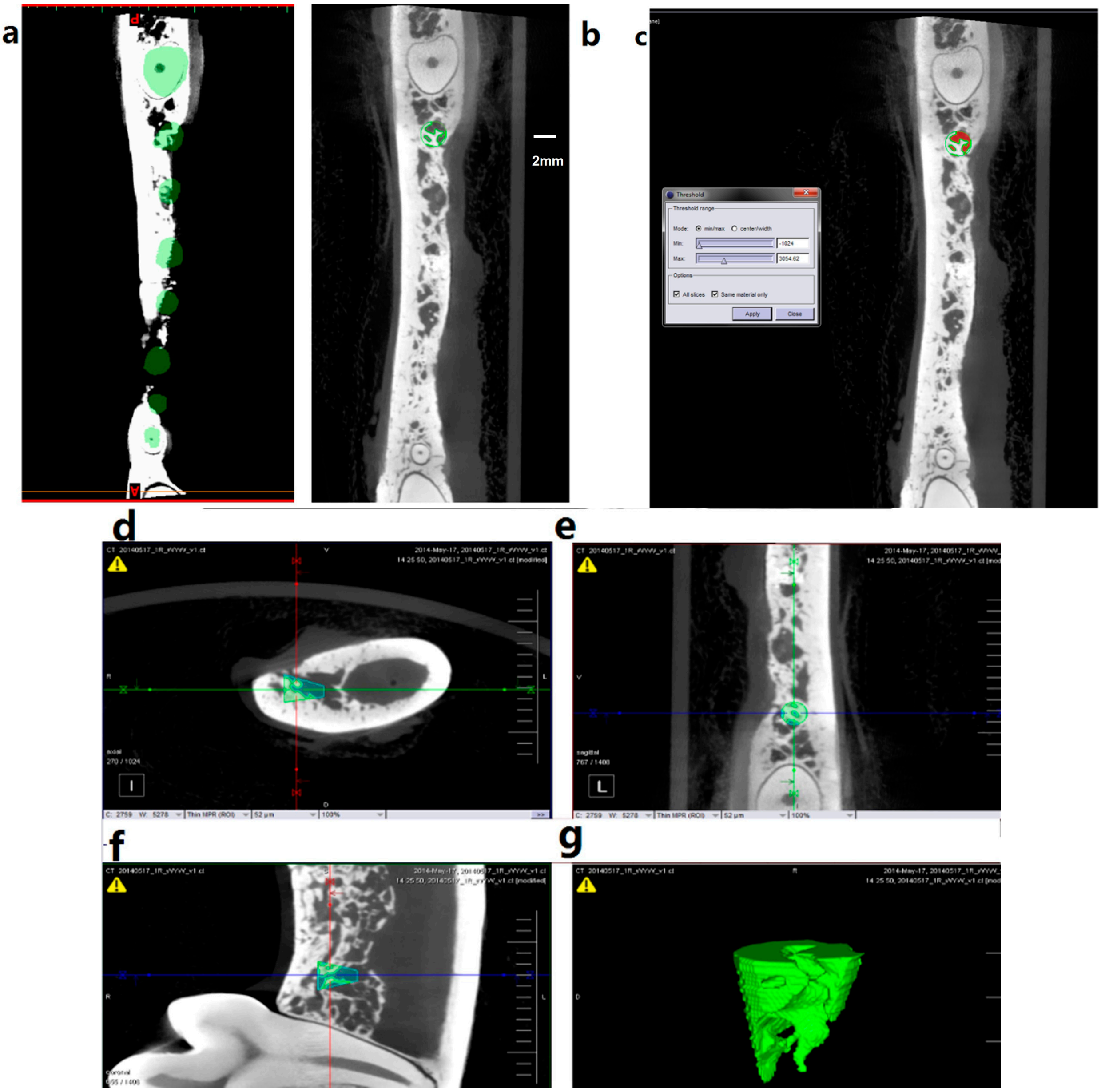

Figure S1. The choosing of ROI: (**a**) the transparency overlay of the superimposed 3D CBCT root model and μCT model to help locate (**b**) the position of ROI; (**c**) the threshold to determine trabecular bone and bone marrow, the threshold was 3054.62 for all specimen. The ROI was a root-shaped region with a 2 and 1 mm diameter on the upper and lower side of alveolar ridge respectively and with a height of 3 mm, shown in (**d**–**f**); (**g**) the 3D reconstruction of trabecular bone.
